# Exploring Environmental Settings to Improve the Printability of Paroxetine-Loaded Filaments by Fused Deposition Modelling

**DOI:** 10.3390/pharmaceutics15112636

**Published:** 2023-11-16

**Authors:** Sara Figueiredo, Ana I. Fernandes, Fátima G. Carvalho, João F. Pinto

**Affiliations:** 1 iMed.Ulisboa—Research Institute for Medicines, Faculdade de Farmácia, Universidade de Lisboa, Av. Prof. Gama Pinto, 1649-003 Lisboa, Portugal; sara.figueiredo@anf.pt (S.F.); jfpinto@ff.ul.pt (J.F.P.); 2 LEF-Infosaúde, Laboratório de Estudos Farmacêuticos, Rua das Ferrarias del Rei nº6, Urbanização da Fábrica da Pólvora, 2730-269 Barcarena, Portugal; fatimag.carvalho@anf.pt; 3 Egas Moniz Center for Interdisciplinary Research (CiiEM), Egas Moniz School of Health & Science, Campus Universitário, Quinta da Granja, 2829-511 Caparica, Portugal

**Keywords:** 3D-printed tablet, desiccator drying, filament, fused deposition modelling (FDM), hot melt extrusion (HME), microwave drying, oven drying, paroxetine (PRX), printability

## Abstract

The successful integration of hot-melt extrusion (HME) and fused deposition modelling (FDM) depends on a better understanding of the impact of environmental conditions on the printability of formulations, since they significantly affect the properties of the raw materials, whose control is crucial to enable three-dimensional printing (3DP). Hence, the objective of this work was to investigate the correlation between the environmental settings and the properties of paroxetine (PRX)-loaded filaments, previously produced by HME, which affect printability by FDM. The influence of different drying methods of the physical mixtures (PMs) and HME-filaments (FILs) on the quality and printability of these products was also assessed. The printability of FILs was evaluated in terms of the water content, and the mechanical and thermal properties of the products. Stability studies and physicochemical, thermal, and in vitro dissolution tests were carried out on the 3D-printed tablets. Stability studies demonstrated the high ductility of the PRX loaded FILs, especially under high humidity conditions. Under low humidity storage conditions (11% RH), the FILs became stiffer and were successfully used to feed the FDM printer. Water removal was slow when carried out passively in a controlled atmosphere (desiccator) or accelerated by using active drying methods (heat or microwave). Pre-drying of the PRX/excipients and/or PMs did not show any positive effect on the printability of the FIL. On the contrary, dry heat and, preferably, microwave mediated drying processes were shown to reduce the holding time required for successful FDM printing, enabling on-demand production at the point of care.

## 1. Introduction

Three-dimensional printing (3DP) is an emerging technology in pharmaceutics with undeniable advantages over traditional manufacturing processes, namely regarding individualization of medicines [1]. Actually, 3DP allows for the production of dosage forms with a specific and precise dose and geometry [2,3], tailored to each patient’s need. Unique 3D-printed medicines may contain multiple drugs [4,5], and/or offer tailored drug release profiles [6,7,8], expanding the applicability of the technology, with remarkable benefits for the patient, including the ability to combine multiple drugs in a single dosage unit and customize drug release, and increase patient compliance, treatment effectivity, and cost-effectiveness [9,10]. In addition, specific age groups (e.g., pediatrics and geriatrics) and/or diseases or medical conditions (e.g., renal or hepatic impairment, oncology, psychiatry) now have the greatest opportunity to maximize therapeutic outcomes through the development and manufacture of customized medicines [11,12].

Fused deposition modelling (FDM), the most commonly used 3DP technique, uses a drug-containing filament, previously obtained by hot-melt extrusion (HME). During HME, both heat and shear are applied to promote the dispersion of the drug(s) in the polymer matrix, with the subsequent production of an extrudate, or filament, in which the drug is predominantly and preferably amorphous [13]. The FDM 3D printer is then fed with the produced filament, which is melted and continuously deposited onto a surface, layer by layer, creating the 3D-printed medicine [11,14,15].

The coupling of HME with FDM has been attempted to produce on-demand dosage forms in a single, or more often sequential, manufacturing process. As a continuous process, it would offer remarkable improvements not only in terms of the manufacturing process (fewer steps, less human error, reduced manufacturing time), but also in terms of the finished pharmaceutical products (less batch-to-batch variation, greater reproducibility, and reliable pharmacological effect), culminating in a more cost-effective and efficient way to produce patient-centric, designed solid dosage forms [16,17,18].

Nevertheless, coupling of HME and FDM technologies depends on the choice of the pharmaceutical polymers, formulation, and process parameters [17,18,19,20]. To be successful, the combination of HME and FDM requires that both the extrudability of the raw materials and the printability of the HME filaments produced are achieved, a goal that is influenced by the mechanical, rheological, and thermal properties of the materials [17,18,19,20]. In fact, the extrudability of a material does not guarantee that the produced filament will behave appropriately during printing [21,22]. To enable 3DP, the raw materials must have specific properties that are suitable for both technologies. For example, continuous operation with high throughput requires adequate feeding of the printer with filaments of the appropriate dimensions [23], stiffness [24,25], and rheology [26], to avoid breakage or coiling during printing, and/or jamming of the printer [27]. In particular, the evaluation of the mechanical and thermal properties of filaments is of paramount importance as the filament is drawn by the printer feeding gears towards the heated nozzle where it softens to allow for the accurate deposition onto the build plate. These properties are influenced not only by the composition of the filament (e.g., polymer matrix, additives, and drug) and the processing parameters used, but also by the filament’s storage conditions [19,20,21,27,28].

In addition, a minimum holding time after filament production is often required for adequate printability [20,21,28,29], making the process unacceptably long for on-demand production. In previous studies, we found that PRX-loaded filaments were not printable immediately after extrusion [20]. In fact, at least one week of storage in a desiccator at low RH (11%) was required for the filaments to exhibit suitable properties for the subsequent printing process. Polymeric filaments were highly ductile immediately after HME, and therefore their remarkable flexibility caused printer feeding defects. These results indicated not only the significant influence of the environmental conditions (particularly moisture) on filament properties, but also the significant impact of these properties on filament printability, which is critical to the success of 3DP. In other works, hydroxypropyl cellulose (HPC)-based filaments, which exhibited high moisture uptake at high relative humidities, also failed to be printable [21]. In this context, water promotes excessive hydration, excessive polymer swelling, and plasticity of the cellulosic polymers, which promotes the formation of a highly viscous and poorly flowing material, ultimately leading to filament coiling or nozzle blockage [28]. For example, Dumpa et al. [29] observed irregular material flow along the printer nozzle as a result of the effect of the moisture content on filament softening and squeezing. The authors suggested storing the filaments in a desiccator prior to printing to minimize the influence of excess water. In another study, the removal of moisture from cellulose filaments by preheating the filaments before printing was suggested [28]. In fact, drying the filaments to control their moisture content and promote the success of FDM processes has been recommended in the studies available in the literature to date [28,29]. Furthermore, the use of dried filaments has been suggested as a way to improve the physical and chemical properties of the intermediate and final products of this manufacturing process. Wang et al. [30] demonstrated that microwave heating improved the mechanical properties of 3D-printed SiC-coated PLA composite parts in terms of tensile strength, Young’s modulus, and interlayer stress at break. Complementary works have previously demonstrated that microwave post-treatment improves the mechanical properties of polymer matrix-based composites [31,32]. In addition, a study by Löbmann and colleagues [33] found that both convection and microwave-induced radiation play a crucial role in promoting in situ drug amorphization, which can influence their bioavailability and clinical effectiveness. Some of these studies have investigated different drying methods, with the expectation that the heating mechanism underlying microwave drying might be more beneficial to the performance and quality of the products [33,34,35,36]. However, the work published to date has focused on the application of the drying step only to intermediate products obtained by HME. The positive impact of this process on the performance and quality of the polymeric products obtained as well as on the total time of the manufacturing process remain unclear.

Therefore, in the present work, materials at different stages of manufacture (single or blended raw materials and filaments) were dried using different methods. To the best of the authors’ knowledge, this is the first time that the effect of moisture on the printability of filaments made from well-accepted materials, has been evaluated in a single, systematically designed study. Specifically, we aimed to investigate the correlation between the environmental conditions (moisture dependence) and the properties of paroxetine (PRX)-loaded filaments manufactured by HME, which affect their printability by FDM, in order to optimize and accelerate integration of HME into FDM, to enable expeditious and timely manufacture of customized solid dosage forms in a manner acceptable to both patients and pharmacists.

## 2. Materials and Methods

### 2.1. Materials

Paroxetine hydrochloride (Eur. Ph., melting point of 121–131 °C, Form II) was supplied by Lusifar (Lisbon, Portugal) and used as a model drug. Hydroxypropylcellulose (HPC^TM^ LF Pharm, donated by Ashland Inc., Schaffhausen, Switzerland), triethylcitrate (TEC, Sigma Aldrich, Darmstadt, Germany), dicalcium dihydrate phosphate (CaP; Budenheim, Rheinstrasse, Germany) and magnesium stearate (MS; Roic Pharma, Terrasa, Barcelona, Spain) were used as excipients. Karl-Fisher titration was performed with Hydranal™- Composite 5 (Honeywell, Charlotte, NC, USA) and the different RH conditions were obtained with lithium chloride (Merck KGaA, Madrid, Spain). All other reagents were of analytical grade.

### 2.2. Production of 3D-Printed Tablets

A physical mixture (PM) of PRX (30% *w*/*w*), HPC (54% *w*/*w*) and other excipients (mixture made of 10% *w*/*w* of CaP, 1% *w*/*w* of MS and 5% *w*/*w* of TEC), as previously established [19], was produced in a mortar (15 min) and sieved (500 µm) to remove agglomerates. The homogeneity of the mixtures was determined by PRX quantification at 294 nm (see Section 2.7) at different points.

**Figure 1:**
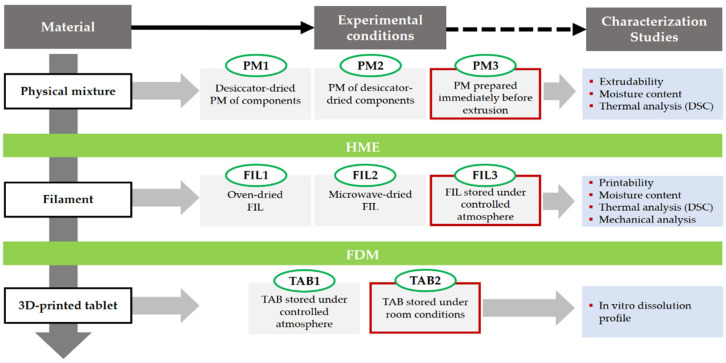
Layout of the experimental design of the work. PRX-based formulations were hot-melt extruded into filaments (FILs), amenable to producing 3D-printed tablets (TAB) by FDM. Physical mixtures of components were (1) stored in a desiccator after mixture (PM1), (2) pre-stored in a desiccator (PM2) and (3) used as received, i.e., not-desiccated (PM3, reference). The filaments obtained by extrusion were (1) dried in a hot-air oven (FIL1), (2) dried in a microwave oven (FIL2) or (3) stored under controlled atmosphere in a desiccator (FIL3, reference). Tablets were stored in a desiccator (TAB1) or kept under room conditions (TAB2). Reference samples are highlighted in red, whereas the characterization studies performed on the various materials are presented in the light blue boxes.

Figure 1 presents an outline of the experimental design used in the work. The different experimental conditions encompassed pre-dried PMs (PM1; after mixture, the PMs were placed in a desiccator at 11% RH for 12 h, before HME) and PMs prepared from pre-dried components (PM2; the drug and excipients were individually placed in a desiccator at 11% RH for 12 h, before HME). The conditions in the desiccator (20 °C/11% RH) were obtained with a saturated solution of lithium chloride [37]. Reference PMs (PM3) were prepared immediately before extrusion from all formulation components stored under room conditions (20 °C/65% RH).

Each PM was extruded in a single-screw extruder (Noztec Pro, Noztek, Shoreham, UK) at temperatures of 120 °C and 90 °C (barrel with two heating sections), at a constant screw speed (10 rpm).

Filaments were dried after processing either in a convection hot-air oven (40 °C/24 h, Thermo-Heraus VT6060P drying oven, Waltham, MA, USA)—FIL1—or in a microwave oven for 45 min (chamber with 315 × 325 × 202 mm at a frequency of 2450 ± 50 Hz; input power 1150 W and output power 700 W, Jocel^®^ D70D17L-D5 Microwave, Santo Tirso, Portugal)—FIL2. FIL3 filaments were maintained under controlled relative humidity conditions (20 °C/11% RH) until printing.

Tablets were printed from PRX-loaded filaments (3D printer Delta WASP 20 40 Turbo 2, Wasp, Massa Lombarda, Italy) according to a digital template (3D Sprint Software v2.11, 3D Systems, Rock Hill, SC, USA) and exported as a stereolithography (.stl) file into Cura (v15.04.2, Ultimaker B.V., Utrecht, The Netherlands). The tablets (cylinders with 10 mm diameter × 3 mm thick; 0.7 mm layer width × 1.4 mm wall thickness; 100% infill) were printed at 200 °C using a 60 mm/s printing speed. Tablets printed were stored under controlled atmospheres (TAB1, 20 °C/11% RH) in a desiccator, or under room conditions (TAB2, 20 °C/65% RH).

The physical mixtures, filaments, and 3D-printed tablets were characterized according to the experimental design highlighted in the light blue boxes (Figure 1). The ‘Supplementary Materials’ section features illustrative images of the filaments and 3D-printed tablets produced in this work.

### 2.3. Stability Studies

To monitor the physical changes of the filaments over time, a study was conducted considering the climatic conditions set by ICH Q1A [38]. The filaments (n = 3) were stored in climatic chambers (Fitoclima D1200PH, Aralab, Albarraque, Portugal) at 25 °C/60% RH, 30 °C/65% RH, and 40 °C/75% RH inside an open plastic bag, and re-examined at pre-defined times (0, 1, 2, 3, 7, and 30 days). Mechanical and thermal properties, moisture content, and feeding/printing performance of the filaments were evaluated for each period time and storage condition.

### 2.4. Mechanical Tests

The mechanical properties of the filaments (n = 3) were evaluated with a texture analyzer, TA-XT plus (Stable Systems, London, UK), equipped with a 3-point bend rig HDP/3PB apparatus (Stable Micro Systems, London, UK), with the leaver rate set at 1 mm/s, for a total displacement of 10 mm and a 30 mm distance between the sample holders, according to ISO 527 (tensile mode) [39]. The determinations were performed in triplicate using 10-cm-long filaments. Attaching the filaments on both sides of the support allowed for easier reading of the deformation profile and greater reproducibility of the results [40]. After drawing a stress-strain curve based on the filament diameter, mechanical parameters, such as the Young’s modulus of elasticity (E), the stress at maximum load (σ), the strain at break (ε), and the strain energy, were calculated. The Young’s modulus was determined according to the following equation:

E = σ/ε(1)

where, σ is the stress at maximum load and ε is the strain at break.

### 2.5. Moisture Content

The moisture content of the filaments (n = 3) was determined using a Mettler Toledo V30 volumetric Karl Fischer titrator (Mettler Toledo, Schwerzenbach, Switzerland). The filaments were fragmented and immediately placed in a glass sample holder. The moisture released from the sample was conveyed to the titration vessel by a nitrogen stream (300 m/min), after which the titration was performed.

### 2.6. Thermal Analysis

Differential scanning calorimetry (DSC) thermograms (TA Instruments calorimeter, Q200, New Castle, DE, USA) of the PMs, filaments, and tablets were obtained to identify the PRX crystallinity state and evaluate the miscibility of the drug in the polymer. The samples (5–10 mg) were placed in hermetically sealed, nitrogen-purged (10 mL/min flow), aluminum pans, and scans (n = 3) were conducted within a temperature range of −65 °C to 250 °C. Data analysis was performed using proprietary software (Universal Analysis 2000, version 4.7A, 2009, TA. Instruments, New Castle, DE, USA).

### 2.7. In Vitro Dissolution Tests

Dissolution tests were carried out using a dissolution apparatus (AT7, Sotax, Aesch, Switzerland) according to the Eur. Ph. 2.9.3 basket method (apparatus I) [41]. Each replicate (n = 3) was conducted at 37.0 °C ± 0.5 °C and 50 rpm, using 900 mL of dissolution medium (HCl 0.1N). Over the course of the 12-h experiment, 5-mL aliquots were periodically collected at 15, 30, 45, 60, 90, 120, 180, 240, 360, 480, and 720 min. No replacement was made for the withdrawn volume and corrections were made for PRX concentration determinations. After collection, each sample was centrifuged at 4400 rpm for 15 min (Mega Star 600R, VWR, Geldenaasksban, Leuven, Belgium). Quantification was performed by UV spectrophotometry (λ = 294 nm, Cary Win UV 60, Agilent Technologies, Santa Clara, CA, USA). The dissolution profiles were characterized by determining t_50%_, i.e., the time required for 50% of the drug to be released, and the dissolution rate (DR), i.e., the slope of the mass released versus time curve, from zero to the eighth time point of analysis. In addition, the similarity factor (f_2_) proposed by the Food and Drug Administration (FDA) was calculated to compare the drug release profiles [42], according to the following equation:

(2) $$f_{2}=50\times log\left\{{\left[1+(\frac{1}{n})\sum_{t=1}^{n}{\left(R_{t}-T_{t}\right)}^{2}]\right]}^{-0.5}\times 100\right\}$$

where, *n* represents the number of time points, *R_t_* denotes the dissolution value of the pre-change reference batch at time *t*, and *T_t_* represents the dissolution value of the post-change test batch at time *t*.

### 2.8. Statistical Analysis

One-way analysis of variance (ANOVA) was used to analyze the results, and when statistical significance was obtained, post hoc Tukey’s test was conducted to compare the drying processes or type of PMs (GraphPad Prism v.9.2.0, San Diego, CA, USA). Statistical significance was considered at *p* < 0.05.

## 3. Results and Discussion

Previous studies have shown that PRX-based formulations can be extruded by HME into filaments, which in turn can be used to feed an FDM printer to produce 3D-printed tablets [19,20]. All the characterization tests conducted on the finished dosage forms met the criteria of the European Pharmacopeia (Eur. Ph.) [41], indicating their potential for clinical application, namely for dose adjustment in psychiatry [43]. Nonetheless, it was established that the PRX-loaded filaments were not immediately printable after the extrusion process [20]. The ability to quickly produce 3D-printed dosage forms on demand, is crucial for the implementation of 3DP in clinical practice; however, the required holding time poses a serious constraint, warranting further investigation to reduce this limitation.

As such, stability studies were conducted on the filaments under various climatic conditions (low and high humidity) to determine the optimal environmental settings for processability. The thermal and mechanical properties, as well as the moisture content of filaments, were assessed and correlated with quality and printability. Optimization studies were also conducted on the integrated HME-FDM manufacturing process. This was improved by including post-processing steps (drying process) for the PMs and filaments, which expedited water loss and achieved optimal product attributes, guaranteeing successful printing and accelerating dosage form production by FDM.

### 3.1. Evaluation of Filament Quality and Printability

Stability studies were conducted to evaluate how environmental settings affect filament quality and printability. To this aim, PRX-based formulations were prepared immediately prior to extrusion (PM3 as Reference).

Only the filaments stored in a desiccator were printable after 1 week of production. On the contrary, the filaments stored in the climatic cabinets (all environmental conditions) lacked adequate mechanical behavior for printer feeding, since they were too pliable (Table 1).

**Figure 2:**
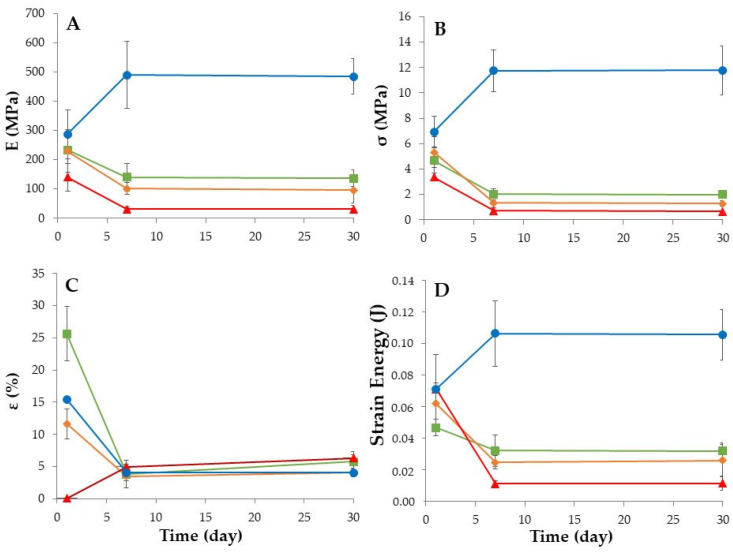
Mechanical properties of the filaments during the stability studies (n = 3): (**A**) Young’s modulus; (**B**) stress at maximum load; (**C**) strain at break and (**D**) energy strain over time and storage conditions (25 °C/60% RH, green line; 30 °C/65% RH, orange line; 40 °C/75% RH, red line; and 20 °C/11% RH, blue line).

Concurrently, the evaluation of the filaments’ performance during printing was correlated with their water content and mechanical properties (Figure 2). The filaments kept in climatic chambers (high humidity, i.e., >60% RH) became more ductile due to moisture absorption. Water promoted plasticization of the filaments, and, as depicted in Figure 2A, significant deformation was necessary for fracture, rendering them unsuitable to feed the printer. The results align with the reduced stiffness and stress observed at maximum load (Figure 2B), during the mechanical tests. The strain energy (Figure 2D) experienced a significant decrease throughout the study, particularly under severe conditions (40 °C/75% RH), as previously reported in the literature [28,29].

**Figure 3:**
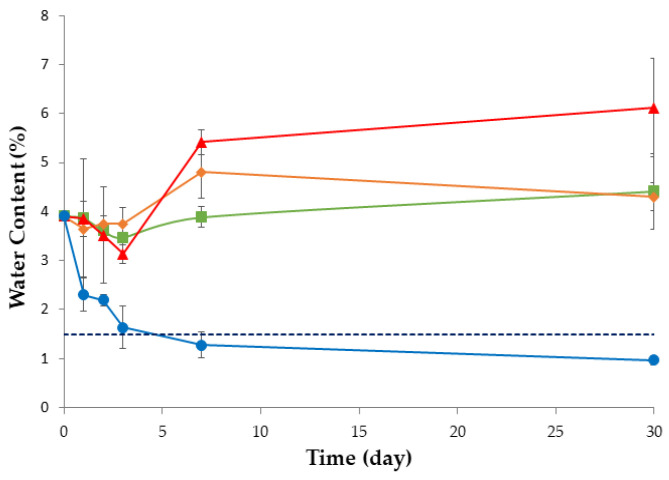
Water content (%) of PRX-based filaments (n = 3) over time and storage conditions (20 °C/11% RH, blue line; 25 °C/60% RH, green line; 30 °C/65% RH, orange line; 40 °C/75% RH, red line). Dashed line represents the threshold above which filaments became pliable.

**Figure 4:**
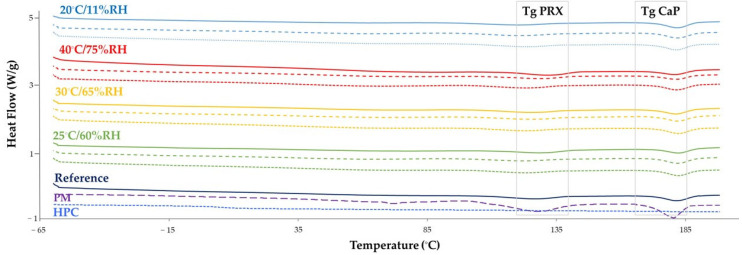
Thermograms of PRX-based filaments (n = 3) over time and storage conditions. Each set of three lines represents the different stability time points [day 1, square dotted line (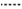); day 7, dashed line (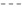); day 30; solid line (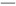)] for a specific storage condition (25 °C/60% RH, green line; 30 °C/65% RH, yellow line; 40 °C/75% RH, red line; and 20 °C/11% RH, blue line). The purple dashed line corresponds to the PM before extrusion, the dark blue solid line corresponds to the filament obtained at day 0 (reference) and the light blue dotted line corresponds to HPC.

These data were also supported by the increase in water content of the filaments during the stability study (all conditions; Figure 3). However, despite water exposure, drug recrystallization did not occur during the study, as confirmed by thermal sample evaluation, which did not show an exothermic event (due to cold recrystallization) besides the endothermic event (due to T_g_; Figure 4). Notably, PRX displayed an endothermic event at 133–135 °C, consistent with previously reported values in the literature regarding thermal analysis of this drug [44]. The thermal event remained stable throughout the study, regardless of the stability condition.

Conversely, storing the filaments at low relative humidity (20 °C/11% RH) caused water loss and proved to be the ideal condition, since this reduced their initial ductility (with an increase in stiffness), enabling proper printer feeding after 1 week of production. The decrease in ductility of the filaments is inferred from both the increase in stiffness and stress under load (with higher strain energy), as well as the decrease in strain at break (Figure 2C).

The results emphasize the crucial role that moisture plays in the mechanical behavior of filaments, which is paramount to successful FDM printing. As previously reported, filaments should not be excessively stiff because they will not bend properly onto spools, nor should they be excessively brittle and prone to breaking when loaded into the printer head [17,18].

### 3.2. Optimization of the Integrated HME-FDM Process

The successful implementation of FDM relies on the ability to manufacture pharmaceutical dosage forms that meet patients’ needs, in a timely manner. Overcoming the technical limitation previously observed is necessary, as 3DP extrusion was only possible one week after the HME process due to the need for the filaments to lose water content to achieve proper mechanical properties. In order to expedite the production of 3D-printed tablets, a set of experiments were conducted that involved drying the raw materials and/or filaments to accelerate water loss. The experimental conditions of the research design were previously outlined in Figure 1.

The various PMs prepared and dried using different methods were extruded into filaments, which were stored under a controlled atmosphere in a desiccator (PM3, reference), or subjected to an additional drying step in a hot-air oven (PM1) or a microwave oven (PM2). The printability, mechanical and thermal properties, and moisture content of the fabricated filaments were evaluated and characterized. The 3D-printed tablets produced were kept under room conditions (TAB2, reference), or stored in a controlled atmosphere (desiccator). The study evaluated the effect of the environmental conditions on the quality and performance of finished products through in vitro dissolution profile assessment.

Drying processes were successfully employed to hasten the continuous HME/FDM manufacturing process. In fact, while the undried filaments were not printable, the filaments that underwent post-processing drying (hot-air oven or microwave oven; FIL1 and FIL2) were more suitable for 3D tablet printing compared with the reference drying method (desiccator; FIL3), provided the filaments were immediately fed into the printer. FDM was performed successfully on the same day (microwave-dried; FIL2) or the following day (oven-dried; FIL1) following HME in the case of active drying processes; when filaments were stored under a controlled atmosphere in a desiccator (FIL3) it took 1 week for successful printing.

#### 3.2.1. Mechanical Studies

**Figure 5:**
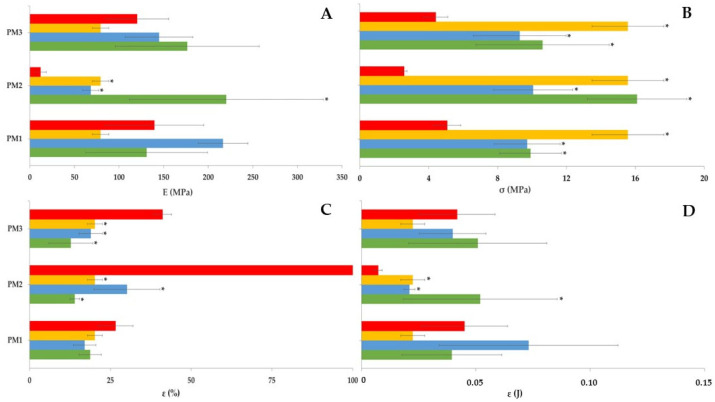
Mechanical properties of filaments (n = 3) prepared from different PMs (dried mixture, PM1; mixture of dried components, PM2; and reference mixture, PM3) when submitted to different drying processes (non-dried FIL, red column; FIL dried in an hot-air oven (FIL1), green column; FIL dried in a microwave oven (FIL2), blue column; and FIL stored in a controlled atmosphere in a desiccator, (reference, FIL3), orange column): (**A**) Young’s modulus; (**B**) stress at maximum load; (**C**) strain at break and (**D**) energy strain over time and storage conditions. * *p* < 0.05 (one-way ANOVA and post hoc Tukey’s test for comparison of the drying process or type of PM).

The mechanical studies conducted on the filaments provided evidence of increased performance after drying. Briefly, a decrease in deformability and an increase in stiffness were observed (Figure 5B,C), as expressed by the higher stress at maximum load (σ) and lower strain at break (ε). In addition, a reduction in plasticity and an increase in elasticity, as indicated by the rise in the Young’s modulus (E) and strain energy (Figure 5A,D) were observed. These results were consistent and independent of the PM or drying method used. Transversely, filaments submitted to a drying step exhibited a reduced ability to withstand stress-induced deformation and were more prone to breaking compared with the non-dried counterparts.

The desiccator-controlled storage of the intermediate products had no substantial effect on the mechanical properties (Young’s modulus of elasticity and energy strain) of the filaments since the changes observed were not statistically significant when compared with the non-dried filaments. On the contrary, drying filaments in a hot-air or microwave oven, had a significantly greater effect (*p* < 0.05, Figure 5B,C) on the mechanical properties (stress at maximum load (σ) and strain at break (ε)) of the dried filaments than the non-dried filaments made. Furthermore, the air-drying oven process had a greater effect on the mechanical properties of the filaments than the microwave oven process. This difference is likely due to the distinct mechanisms of drying. During the air oven drying process, energy is conducted from the surface of the filament to the core for heat transfer, while in the microwave oven drying process, heat is transferred from the core to the surface of the filaments due to dipole formation in molecules. This results in a smaller heat gradient and a more uniform temperature throughout the filament structure. In this regard, microwave-heated filaments undergo more uniform physical transformations due to heat compared with air-dried filaments. The thermal and humidity gradients occur in the same direction in microwaving, unlike the convective oven-based drying process, which causes both surface and internal moisture to start drying immediately after the process commences [34,35]. Consequently, the high drying rate and low drying time associated with the microwave-based process reflect the mechanism of heat transfer.

Dried mixture components (PM2) had a greater impact on the mechanical properties of the filaments, as evidenced by the more significant changes in all the mechanical parameters (*p* < 0.05, Figure 5A). This suggests that pre-drying individual components (PRX and excipients) may influence the establishment of interactions between the components and, consequently, their miscibility during extrusion, affecting the mechanical behavior of the produced filaments. In contrast, the dried mixture (PM1) and reference (PM3, i.e., prepared immediately before the extrusion process) did not exhibit any statistically significant differences in the mechanical properties of the filaments obtained after the drying procedure, in comparison with non-dried filaments.

Overall, these findings suggest that drying of the blend components prior to HME and the drying of the filaments in a hot-air oven have the most significant effect on the mechanical properties of the intermediate products. Since the post-processing drying step is expected to promote minimal changes in the filaments’ printability, it is anticipated that no prior pre-drying of the raw materials and microwave drying of the filaments could be worthwhile strategies to accelerate the integrated manufacturing process, without causing significant polymeric structural changes in the filaments.

Previous studies have demonstrated that microwave-based post-treatment of polymer matrix-based composites enhanced tensile strength and minimized defects compared with air oven drying [31,32]. Moreover, microwave oven drying is more efficient in terms of time and energy, making it a promising option to hasten printability after HME [32].

#### 3.2.2. Moisture Content

**Figure 6:**
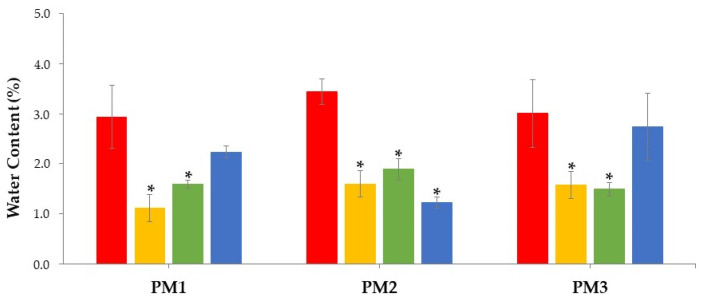
Water content of filaments (n = 3) prepared from different PMs (dried mixture, PM1; mixture of dried components, PM2; and reference mixture, PM3) once submitted to different drying processes (undried FIL, red column; FIL dried in a hot-air oven (FIL1), green column; FIL dried in a microwave oven (FIL2), blue column; and FIL stored in a controlled atmosphere in a desiccator, (Reference, FIL3), orange column). * *p* < 0.05 (one-way ANOVA and post hoc Tukey’s test for comparison of the drying process or type of PM).

As expected, the drying processes effectively decreased the water content of the filaments, leading to statistically significant results compared with the non-dried filaments (*p* < 0.05, see Figure 6). However, there were two exceptions when the drying process was performed in a microwave oven: filaments consisting of pre-dried component blends (PM1) and the reference PM, which maintained levels of moisture comparable to the non-dried filaments, although not affecting printability.

Overall, microwave drying resulted in greater water content than hot-air drying, under the tested instrumental conditions. In future studies, it might be worthwhile to investigate if prolonging the drying period at lower microwave potency (to prevent FIL burning) lowers water content to levels comparable to those of hot-air drying. As reported previously, filaments dried in a hot-air oven presented less variable properties, compared with those dried in a microwave oven [31,35].

#### 3.2.3. In Vitro Dissolution Tests

As an antidepressant drug used in psychiatric treatment [43], it is crucial for PRX to achieve extended drug release to ensure the pharmacological effect without requiring frequent administration. In this context, the dissolution profile and kinetic parameters (t_50%_, DR and f_2_ factor) provide in-depth characterization of the in vitro performance of the 3D-printed tablets obtained.

**Figure 7:**
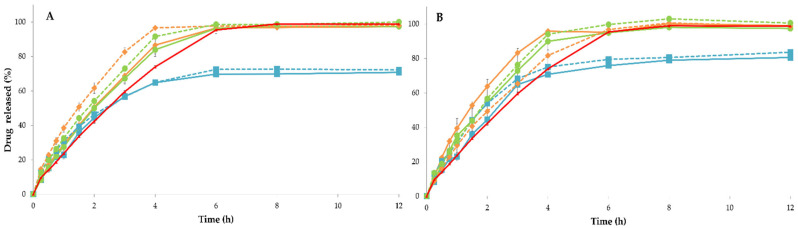
Dissolution profiles of printed PRX tablets (n = 3) (TAB stored in a controlled atmosphere (desiccator, TAB1), solid line; and TAB kept under room conditions (TAB2); dashed line), in which the filaments were submitted to drying process in (**A**) hot-air oven and (**B**) microwave oven, before FDM (dried mixture (PM1), blue line; mixture of dried components (PM2), orange line; and reference mixture (PM3), green line). The reference (red line) corresponds to drug release profile obtained for 3D-printed tablets manufactured from the filaments composed of the reference PM3 and stored in a controlled atmosphere (desiccator; TAB1).

As previously reported, after a certain time, drug release rates reach a plateau for PRX-based formulations [20]. This work shows that under most experimental conditions, at least 85% of PRX was released within the first 4–6 h, and the release reached completion (≈100%) by 6–8 h (Figure 7).

Regardless of the drying method used, the dissolution profile of the dried component mixture (PM2) showed a faster release of PRX (with low t_50%_ and high DR) compared with the drug release profile of the dried mixture (PM1) and the reference (PM3; see Figure 7A,B). The interaction between the components of the dried mixture (PM2) was assumed to be insignificant, due to previous loss of water molecules. As a result, PRX release from anhydrous filaments seemed to be facilitated upon contact with the dissolution medium. These results align with the mechanical properties (e.g., Young’s modulus and energy strain), which significantly differed (*p* < 0.05, Figure 5A,D) from those observed in the reference PM3 filaments, which were produced just before extrusion.

In contrast, the filaments manufactured from the dried mixture (PM1) blends as a starting material for HME had a negative impact on the fraction of dissolved drug in the dissolution medium. In fact, the 3D-printed tablets produced from the dried mixture exhibited incomplete PRX release (<80% of PRX dissolved after 12 h of testing) compared with the other experimental conditions (f_2_ ≈ 50, Table 2). This consistent pattern occurred regardless of the drying method used.

Regardless of the PM used as the raw material, the dosage forms printed from microwave-dried filaments (FIL2) (Figure 7B) tended to exhibit a higher rate and extent of PRX release when compared with tablets fabricated from filaments that were stored in a desiccator (FIL3), or products dried in a convection hot-air oven (FIL1) (Figure 7A). This is confirmed by the lower t_50%_ (e.g., a decrease of ≈24% when compared with the standard procedure) and higher DR values (e.g., increase up to a maximum of 40% when compared with the standard procedure), as presented in Table 2. Despite the higher values, PRX release kinetics appeared to be more variable under microwave drying of the filaments, in line with the mechanical properties and water content determinations, as previously discussed.

Furthermore, oven drying demonstrated a favorable effect on drug release, when compared with the dissolution profile obtained from FIL3 tablets. This indicates that the drying process plays a key role in PRX dissolution, as illustrated in Figure 7A.

The storage of printed tablets under room conditions can also influence the extent of PRX dissolution. For both drying methods evaluated, storage under room conditions (TAB2) seemed to increase the rate and amount of drug release in comparison with storage in a desiccator, except for tablets manufactured from microwave-dried filaments composed of mixtures of dried starting materials (PM1; FIL2). Therefore, drying the finished dosage forms in a controlled environment (desiccator) after the 3DP process did not appear to provide significant benefits, since 3D-printed tablets stored under room conditions exhibit marginally greater release rates (although not statistically significant).

#### 3.2.4. Thermal Analysis

**Figure 8:**
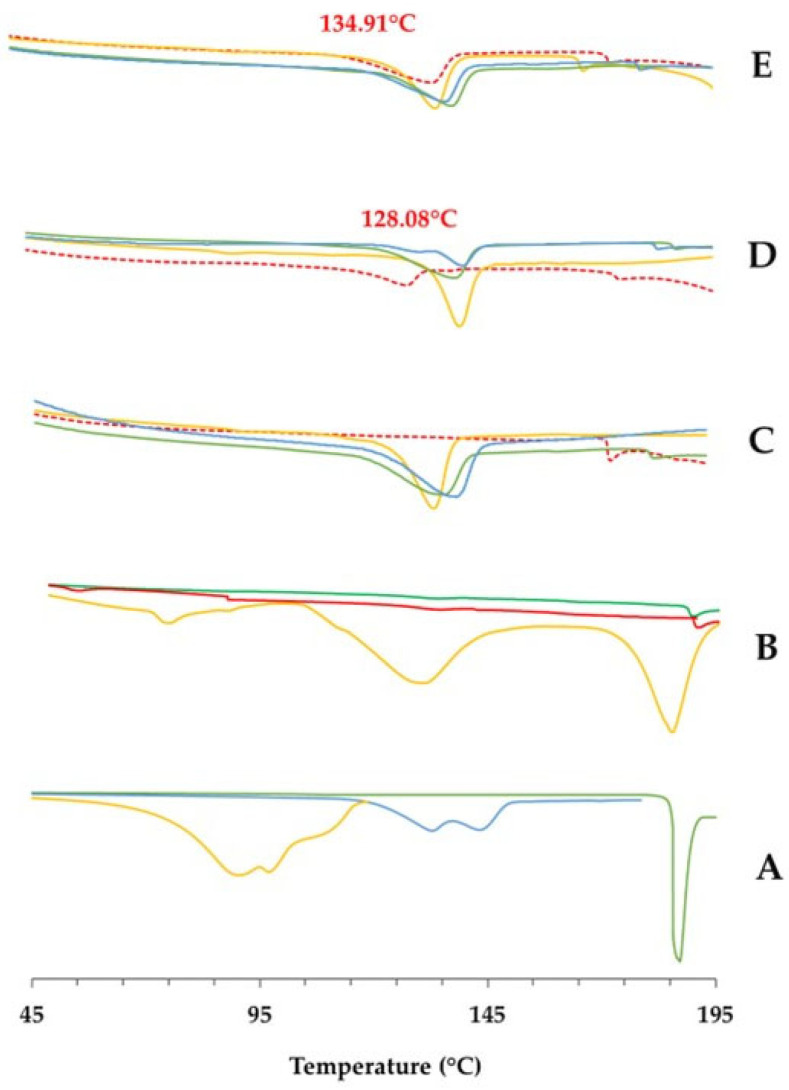
Thermograms of (**A**) PRX-based formulation components (PRX, blue line; CaP, green line; MS, orange line); (**B**) PRX-based products obtained under standard processing conditions: PM prepared immediately before the HME (PM3), orange line; FIL stored in a controlled atmosphere (FIL3), green line; TAB stored under room conditions (TAB2), red line; and PRX-based products obtained after the pre-treatment of PM; (**C**) Desiccator-dried mixture of components (PM1, red dashed line): FIL dried in a hot-air oven (FIL1), solid green line; FIL dried in a microwave oven (FIL2), solid blue line; and FIL stored in a controlled atmosphere in a desiccator (Reference, FIL3), solid orange line; (**D**) Mixture of desiccator-dried components (PM2, red and dashed line): FIL dried in a hot-air oven (FIL1), solid green line; FIL dried in a microwave oven (FIL2), solid blue line; and FIL stored in a controlled atmosphere in a desiccator (reference, FIL3), solid orange line; (**E**) Reference mixture prepared immediately before extrusion (PM3, red dashed line): FIL dried in a hot-air oven (FIL1), solid green line; FIL dried in a microwave oven (FIL2), solid blue line; and FIL stored in a controlled atmosphere in a desiccator (Reference, FIL3), solid orange line.

In this section, thermograms are presented for different PMs (desiccator-dried mixture of components; PM1; mixture of desiccator-dried components, PM2; and reference mixture prepared immediately before extrusion, PM3) and filaments (FIL dried in a hot-air oven, FIL1; FIL dried in a microwave oven, FIL2; FIL stored under controlled atmosphere in a desiccator, FIL3), to evaluate the impact of the post-processing drying on the thermal properties of these materials (Figure 8C,D,E). Figure 8A,B display thermograms of PRX-based formulation components and products, providing further insight into the aforementioned thermograms.

The thermograms of both PMs and filaments exhibited the typical PRX endothermic peak between 128–140 °C, supporting the findings reported by Pina et al. [44], i.e., the conversion of Form II into Form I in the presence of residual water in the raw material. These results demonstrate that extrusion (HME) was able to promote drug amorphization, although incompletely. Furthermore, the data imply that drying of the PM and/or filaments to expedite production can trigger thermal changes and potentially modify the solid state of the drug. Amorphization is unlikely to significantly affect the solubility of PRX, since PRX HCl is described to be slightly water soluble and, at the experimental pH 1–2 used for dissolution, the drug was fully ionized [45]. Furthermore, the absence of PRX endothermal in 3D-printed tablets and dissolution data indicate adequate PRX release.

The thermal behaviors described showed no deviations, except for the dried mixture (PM1), where no endothermic peak related to PRX was found in the thermogram. Interestingly, in the next manufacturing stage, there might have been a compromise in stabilizing the amorphous systems, since the thermograms of filaments obtained from the dried mixture exhibited PRX endotherms (Figure 8C). These data suggest an increased instability within the system, which together with the less satisfactory results from the dissolution test, further emphasize that drying the PM before extrusion is not a promising approach.

Furthermore, for the previously dried filaments, the endothermic transition of drug tended to occur at higher temperatures (Figure 8D,E) compared with the non-dried filaments, as a consequence of the removal of water from the blends. A previous study reported a similar finding, where the thermogram of pure PRX displayed two endothermic events, which could be attributed to dehydration followed by the melting of the drug (Form I) [44].

Likewise, the endothermic peak of PRX presented a wider shape in dried filaments as opposed to the non-dried filaments. Additionally, the thermograms of previously dried filaments showed a broadening and decreased intensity of peaks, likely due to the interaction between formulation components, when compared with PM. These findings suggest that PRX was dispersed among other components, originating a low-intensity endothermic event [44].

The thermograms of the filaments subjected to the microwave drying process (FIL2) revealed a PRX endothermic peak at a higher temperature compared with the oven-dried filaments (FIL1). This difference was more noticeable for filaments composed of a dried mixture (PM1), which had T_g_ values ranging between 134.31 °C (oven drying) and 139.23 °C (microwave). These data seem to be correlated with the mechanism of water removal from filaments (microwave versus air drying in the ovens).

No significant differences were observed in the thermograms of the filaments prepared from different PMs. In certain cases, an endothermic transition occurring at around 190 °C was identified, and associated with the presence of the calcium phosphate in the formulation, as previously reported in the literature [46]. Similarly, the raw material also presented endothermic events at around 90 °C, consistent with the melting of magnesium stearate [47].

Based on the comprehensive analysis of the results, summarized in Table 3, the current investigation suggests that the strategy of pre-drying raw materials (either alone or after blending) did not favor processability. Instead, conducting a preliminary drying of filaments using a microwave oven or hot-air oven can contribute to optimizing the integration of HME-FDM manufacturing. Although both methods show potential, the intervention using microwaves appears to be the most promising, but further research is needed.

## 4. Conclusions

This study confirms that environmental conditions (especially humidity) significantly influence the integration of HME and FDM. Humidity affects the moisture content and mechanical properties of the filaments, and ultimately, printability. In fact, the HME-produced PRX-loaded filaments were highly ductile when exposed to high humidity levels, precluding printing of filaments into tablets. On the other hand, the filaments kept under low humidity conditions were the most suitable for printing PRX tablets. This was due to the water loss that increased the stiffness of the filaments, allowing them to be fed into the printer seamlessly without coiling. Despite the success of the 3DP process, one week was required for the filaments to become printable by FDM. The mechanical properties of the filaments were significantly affected by moisture content, while its impact on the thermal properties was not apparent, as the thermograms did not exhibit substantial differences across humidity conditions.

Addition of a drying step to the manufacturing process expedited tablet printing without compromising their performance. In this respect, although the previous drying of the PM raw materials was not relevant for this specific formulation, drying the filaments can be advantageous. Among the experimental conditions tested, microwave-mediated drying appears to offer the greatest benefit, as it streamlines the 3DP process, allowing it to occur within one day after HME, while also not compromising the dissolution of the PRX tablets (which was in fact promoted, as evidenced by the mean increase of 24% for t_50%_ and decrease of a maximum of 40% for DR values when compared with the standard procedure). However, this drying method resulted in greater variability than hot-air oven drying, affecting not only PRX dissolution, but also mechanical and thermal properties, thus requiring well-controlled instrumental conditions.

Complementary studies, to determine if this behavior is shared by other formulations or drugs with remarkably different properties, are warranted.

## Figures and Tables

**Table 1:** Printability of filaments over time and storage conditions.

Stability Condition (Temp./Humidity)	Time (Day)			
	0	1	7	30
20 °C/11% RH	No	No	Yes ^1^	Yes ^1^
25 °C/60% RH	No	No	No	No
30 °C/65% RH	No	No	No	No
40 °C/75% RH	No	No	No	No

^1^ Printing temperatures of 200 °C (nozzle)/50 °C (plate) were considered in successful printing.

**Table 2:** Dissolution parameters of 3D-printed tablets produced by coupled HME-FDM.

Experimental Settings			T_50%_ (min)	DR (mg.min^−1^)	f_2_	Similarity
Filament Drying	PM Storage	Printed Tablet Storage				
No drying	PM3	Room conditions	147.00	0.203	---	---
Hot-air *	PM1	Desiccator	146.93	0.164	51.56	Yes ^1^
		Room conditions	141.56	0.253	51.06	Yes ^1^
	PM2	Desiccator	118.02	0.160	58.21	Yes ^1^
		Room conditions	88.194	0.275	40.26	No
	PM3	Desiccator	120.08	0.160	62.36	Yes
		Room conditions	107.66	0.240	49.50	No
Microwave **	PM1	Desiccator	143.38	0.164	57.20	Yes ^1^
		Room conditions	108.07	0.283	52.22	Yes ^1^
	PM2	Desiccator	82.385	0.213	39.47	No
		Room conditions	122.726	0.172	63.61	Yes
	PM3 **	Desiccator	107.667	0.191	50.00	Yes ^1^
		Room conditions	104.477	0.255	47.06	No

^1^ Close to 50 (criterion defined for f_2_). Dissolution parameters of printed PRX tablets (TAB stored under controlled atmosphere (desiccator, TAB1); and TAB kept under room conditions (TAB2)), in which the filaments were prepared from PMs (desiccator-dried mixture (PM1); mixture of desiccator-dried components (PM2); and reference mixture (PM3), and then, were submitted to drying process in a hot-air oven and microwave oven, before FDM. * *p* < 0.05; ** *p* < 0.01 (one-way ANOVA and post hoc Tukey’s test for comparison between tablets manufactured from different dried components).

**Table 3:** Summary of the work outcomes according to the experimental design considered.

Experimental Settings			Outcomes (HME/FDM Processability/Quality of FIL and TAB)
PM Storage	Filament Drying	Printed Tablet Storage ^3^	
PM1	FIL1	TAB1	Poor HME/FDM integration strategy ^2^ FIL with increased thermal instability TAB with reduced drug release.
		TAB2	
	FIL2	TAB1	
		TAB2	
	FIL3	TAB1	
		TAB2	
PM2	FIL1	TAB1	Poor HME/FDM integration strategy ^2^ FIL with increased mechanical instability TAB with increased drug release
		TAB2	
	FIL2	TAB1	
		TAB2	
	FIL3	TAB1	
		TAB2	
PM3	FIL1	TAB1	Good HME/FDM integration strategy ^1^ Holding time before HME—2 days FIL with improved mechanical properties and increased water content TAB with decreased drug release rate in comparison with reference More reproducible than microwave oven
		TAB2	
	FIL2	TAB1	Good HME/FDM integration strategy ^1^ Holding time before HME—1 day FIL with reduced mechanical properties and water content TAB with increased drug release rate in comparison with reference Less reproducible than hot-air oven
		TAB2	
	FIL3	TAB1	Reference Holding time before HME—7 days
		TAB2	

Physical mixtures (PM) of PRX-based formulations were hot-melt extruded into filaments (FIL), amenable to produce 3D-printed tablets (TAB) by FDM. PM of components were (1) stored in a desiccator after mixture (PM1), (2) stored in a desiccator before mixture (PM2) and (3) used non-desiccated (PM3, reference). The extruded filaments were (1) dried in a hot-air oven (FIL1), (2) dried in a microwave oven (FIL2) or (3) stored in a controlled atmosphere in a desiccator (FIL3, reference). Tablets were stored in a desiccator (TAB1) or kept under room conditions (TAB2, reference). ^1^ When it was possible to carry out both extrusion processes without significant polymeric structural changes to the FIL and TAB fabricated, the strategy was considered good for optimizing the combined HME/FDM manufacturing process; ^2^ In case optimization was not achieved, the most impacted quality and performance-indicating variables are outlined in the table; ^3^ No significant impact on the quality attributes of the 3D-printed tablets manufactured, regardless of the drying process of PMs and FILs, was observed.

## Data Availability

Data are contained within the article and Supplementary materials.

## Supplementary Materials

The following supporting information can be downloaded at: https://www.mdpi.com/article/10.3390/pharmaceutics15112636/s1, Figure S1: Representative images of filaments dried (A) in a hot-air oven (FIL1) and (B) in a microwave oven (FIL2) or (C) stored in a desiccator (FIL3, Reference); Figure S2: Illustrative images of the 3D-printed tablets (A) stored in a desiccator (TAB1) or (B) kept under room conditions (TAB2).
