# Supplementary material for: Exploring Environmental Settings to Improve the Printability of Paroxetine-Loaded Filaments by Fused Deposition Modelling
Source: Pharmaceutics. 2023 Nov 16;15(11):2636. doi: 10.3390/pharmaceutics15112636 (PMC10675712; doi:10.3390/pharmaceutics15112636)
Supplement: Supplementary file 1 [file pharmaceutics-15-02636-s001.zip › pharmaceutics-2591411-supplementary.pdf]

Supplementary Material

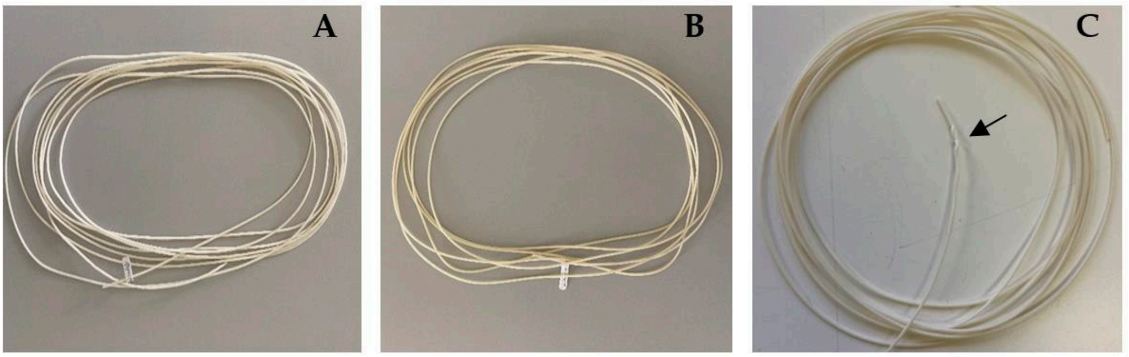

Figure S1. Representative images of filaments dried **(A)** in a hot-air oven (FIL1) and **(B)** in a microwave oven (FIL2) or **(C)** stored in a desiccator (FIL3, Reference).
FIL3 could be printed for up to one week of production, since it was too pliable, causing printer defects (indicated by the arrow in a filament used to feed the printer). FIL1 and FIL2 were obtained from physical mixtures prepared immediately before HME (without drying), and both were printable after oven and microwave drying, respectively.

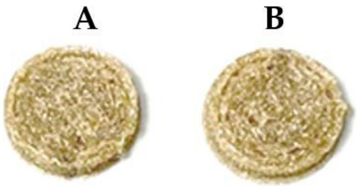

Figure S2. Illustrative images of the 3D-printed tablets **(A)** stored in a desiccator (TAB1) or **(B)** kept at room conditions (TAB2). Both tablets were obtained from physical mixtures prepared immediately before HME (without drying), and filaments dried in a microwave oven. No significant differences were found between tablets stored at room conditions or controlled atmosphere (desiccator).
